# Plasma-Based Synthesis and Modification of Nanomaterials

**DOI:** 10.3390/nano9020278

**Published:** 2019-02-16

**Authors:** Pawel Pohl

**Affiliations:** Division of Analytical Chemistry and Chemical Metallurgy, Faculty of Chemistry, Wroclaw University of Science and Technology, Wyspianskiego 27, 50370 Wroclaw, Poland; pawel.pohl@pwr.edu.pl; Tel./Fax: +48-72-3202494

This Special Issue of Nanomaterials, including nine original research works [1,2,3,4,5,6,7,8,9], is devoted to the application of different atmospheric pressure (APP) and low-pressure (LPP) plasmas for synthesis or modification of various nanomaterials (NMs) of exceptional properties. This is followed by their structural and morphological characterization and further interesting and unique applications in different areas of science and technology. All readers interested in the capabilities of plasma-based treatments will quickly be convinced that APPs and LPPs enable one to efficiently synthesize or modify differentiated NMs using a minimal number of operations. Indeed, the procedures described in the collected articles are eco-friendly and usually involve single-step processes, thus considerably lowering labor investment and costs. As a result, the production of new NMs and their functionalization is more straightforward and can be carried out on a much larger scale, compared to other methods and procedures involving complex chemical treatments and processes. The size and morphology, as well as structural and optical properties, of resulting NMs are tunable and tailorable. In addition to leading to desirable and reproducible physical dimensions, crystallinity, functionality, and spectral properties of the resultant NMs, another benefit of plasma-based synthesis and modification is that fabricated NMs are ready-to-use prior to their specific applications, without any initial pre-treatments.

Among the discharges and plasmas applied for plasma-mediated synthesis or modification of NMs, the readers can find, for example: impulse plasma in a solution initiated by spark discharge between two metallic electrodes immersed in this solution [1]; atmospheric pressure plasma jets provided by dielectric barrier discharge (DBD) operated in Ar–H_2_ [2] or Ar–O_2_, Ar–N_2_, and Ar–NH_3_ [5] mixtures; atmospheric pressure glow discharges (APGDs) generated in air between solid metallic electrodes and flowing solutions [3,4,6]; low-pressure capacitively coupled plasma (CCP) sustained in O_2_ or N_2_ between two electrodes, one being at the end of a chamber field with ionic liquids or low boiling point solvents [7]; and contact glow discharge electrolysis (CGDE) [8] or liquid phase plasma (LPP) [9], operated in both cases between two electrodes immersed in solutions of different compositions. Plasma-chemical processes and reactions occurring directly in plasmas or at interfacial zones between gaseous phases of these plasmas and liquids led to the fabrication of various metal-, nonmetal- and carbon-based NMs, including bimetallic Pd–Fe nanoparticles (NPs) formed by melting and eroding Pd–Fe electrodes [1], fructose-functionalized AgNPs [3], PVP-stabilized PtNPs [4], and pectin-stabilized AgNPs [6] (all synthesized by the reduction of appropriate ions of these metals dissolved in solutions), carbon dots (CDs) formed by irradiation of aliphatic acids dispersed in viscous media, SiNPs fabricated by melting and eroding Si electrodes under plasma heat [8], and nanocomposites supported by Fe_3_O_4_ NPs on N-doped activated carbon [9]. Interestingly, NMs were also synthesized by introducing suspensions of substrates into a plasma torch (suspension-plasma spray, SPS) [2] to form Co/C, Fe/C, and Co–Fe/C NMs, containing a nanometallic phase in addition to carbide and oxide phases of Co and Fe. Immersing plasma jets in liquid suspensions, it was possible to modify the surface of dispersed nanocellulose (NC) fibers [5].

The morphological, structural, and functional properties of NMs result in their having a wide variety of applications, e.g., as catalysts for the Fischer–Tropsch synthesis of CH_4_ from H_2_ and CO [2], antimicrobial agents against different phytopathogenic bacteria [3], heat conductive media in heat management systems [4], necrotic agents toward cancerous cells of the human melanoma cell line [6], fluorescence sensors for detecting and measuring metal ions and flavonoids in solutions [7], and materials for the production of anodes in lithium-ion batteries [8] or electrochemical capacitor electrodes [9].

I sincerely thank all of the authors who sent their valuable works to this Special Issue on Plasma-Based Synthesis and Modification of Nanomaterials. All interested readers are encouraged to become familiar with these works and use discharges and plasmas in their future research on plasma-mediated synthesis or functionalization of nanomaterials.
